# The Benefits of an Augmented Reality Magic Mirror System for Integrated Radiology Teaching in Gross Anatomy

**DOI:** 10.1002/ase.1864

**Published:** 2019-02-19

**Authors:** Felix Bork, Leonard Stratmann, Stefan Enssle, Ulrich Eck, Nassir Navab, Jens Waschke, Daniela Kugelmann

**Affiliations:** ^1^ Chair for Computer Aided Medical Procedures and Augmented Reality, Faculty of Informatics Technical University of Munich Munich Germany; ^2^ Chair for Vegetative Anatomy, Faculty of Medicine Ludwig‐Maximilians University Munich Germany

**Keywords:** gross anatomy education, radiology education, undergraduate education, spatial understanding, anatomy curriculum, clinical anatomy, augmented reality, novel teaching modalities

## Abstract

Early exposure to radiological cross‐section images during introductory anatomy and dissection courses increases students’ understanding of both anatomy and radiology. Novel technologies such as augmented reality (AR) offer unique advantages for an interactive and hands‐on integration with the student at the center of the learning experience. In this article, the benefits of a previously proposed AR Magic Mirror system are compared to the Anatomage, a virtual dissection table as a system for combined anatomy and radiology teaching during a two‐semester gross anatomy course with 749 first‐year medical students, as well as a follow‐up elective course with 72 students. During the former, students worked with both systems in dedicated tutorial sessions which accompanied the anatomy lectures and provided survey‐based feedback. In the elective course, participants were assigned to three groups and underwent a self‐directed learning session using either Anatomage, Magic Mirror, or traditional radiology atlases. A pre‐ and posttest design with multiple choice questions revealed significant improvements in test scores between the two tests for both the Magic Mirror and the group using radiology atlases, while no significant differences in test scores were recorded for the Anatomage group. Furthermore, especially students with low mental rotation test (MRT) scores benefited from the Magic Mirror and Anatomage and achieved significantly higher posttest scores compared to students with a low MRT score in the theory group. Overall, the results provide supporting evidence that the Magic Mirror system achieves comparable results in terms of learning outcome to established anatomy learning tools such as Anatomage and radiology atlases.

## Introduction

In today’s digitized healthcare domain, medical images are more relevant than ever before. Rapid technological advances in both hardware and software have led to an unprecedented surge in the amount of medical data collected, with an estimated size of 2.5 zetabytes by the year 2020 (Hersh et al., 2011). Medical images are not only an important pillar of diagnosis, treatment planning, and follow‐ups, but with the growing field of computer‐assisted surgery, more and more interventions are performed under image guidance. Furthermore, picture archiving and communication systems allow for effortless transmission and permanent availability of this data, such that access is no longer limited to radiologists. A general understanding of different imaging modalities as well as basic image interpretation skills are becoming increasingly important also for non‐radiologists (Orsbon et al., 2014; Zwaan et al., 2017).

The growing relevance and omnipresence of medical images therefore requires increased educational activities to exploit recent developments to appropriately prepare medical students for their future practice. However, recent studies elucidate how today’s radiological education is still lacking at many different levels; limited overall teaching time and large lapses between anatomical and radiological education are main areas for improvement and innovation (Saha et al., 2013; Straus et al., 2014; Heptonstall et al., 2016). Integrating radiology into preclinical anatomy and dissection courses is recognized as an effective avenue to achieve early exposure to medical images while simultaneously increasing student’s motivation and understanding of both radiology and gross anatomy (Murakami et al., 2014; Naeger et al., 2014; Murphy et al., 2015; Grignon et al., 2016; Heptonstall et al., 2016; Sheikh et al., 2016; Paech et al., 2017).

Various integration approaches for teaching radiology and gross anatomy have been proposed in the past, including traditional lectures on interventional radiology (DePietro et al., 2017), cross‐section images on nearby monitors or handheld devices during dissection courses (Lufler et al., 2010; Murakami et al., 2014), peer‐to‐peer interactions with free medical image viewer software (Wilson et al., 2018), e‐learning platforms (Colucci et al., 2015; Mathiowetz et al., 2016; Salajegheh et al., 2016; Darras et al., 2017), and virtual dissection tables such as the Anatomage (Custer and Michael, 2015; Paech et al., 2017, 2018). Ubiquitously, all recommendations favor active learning and suggest a paradigm shift toward multimodal teaching environments (Sugand et al., 2010; Singh and Kharb, 2013; Estai and Bunt, 2016; Phillips et al., 2018).

In recent years, both augmented reality (AR) and virtual reality (VR) have emerged as novel technologies for enhancing educational environments, offering completely new ways for interactive, student‐centered learning (Cheng and Tsai, 2013; Diegmann et al., 2015; Akçayır and Akçayır, 2017). Both AR and VR are two types of mixed reality according to Milgram’s reality‐virtuality taxonomy (Milgram et al., 1995). The taxonomy was later extended by Mann (2002) who introduced the notion of “mediated reality” as a continuum including both the amount of virtuality and mediality. While AR superimposes computer‐generated objects seamlessly onto the user’s view of the real world, VR completely immerses the user in a simulated virtual environment (Azuma, 1997; Azuma et al., 2001).

Several VR systems have been proposed in the past for anatomy education (Marks et al., 2017; Seo et al., 2017; Dominguese et al., 2018). While these systems demonstrated their potential in terms of positive student perception for specific anatomical topics, they were not integrated into larger gross anatomy educational settings. A detailed survey about VR‐based anatomy education systems has been published recently by Preim and Saalfeld (2018). A comparison of mobile and desktop‐based VR systems for learning physiology and anatomy for laryngoscopy was published by Birt et al. (2018). Both the learners’ motivation and skills were found to improve during an undergraduate university course and mobile VR systems were favored compared to more expensive desktop‐based solutions. AR systems for anatomy education are still in its infancies. Several prototypes have been developed and studied with respect to their potential benefits for students (Kiourexidou et al., 2015; Küçük et al., 2016; Wang et al., 2016; Jain et al., 2017; Manrique‐Juan et al., 2017). Hackett and Proctor surveyed three‐dimensional displays and concluded that AR displays can have a positive impact on anatomy education (Hackett and Proctor, 2016). Moro et al. (2017) compared the effectiveness of AR anatomy learning in comparison to both superimposed VR and learning by means of a tablet device. AR was found to be an effective supplement which increased learners’ engagement and motivation. Chien et al. (2010) proposed and evaluated an AR system for interactive learning of structural information about the human skull. While these works present important steps first toward studying the effectiveness of AR systems for gross anatomy education, their quantitative learning effect and their benefits on students’ performance during large scale, curricular gross anatomy courses have yet to be demonstrated.

### Augmented Reality Magic Mirrors

**Figure 1:**
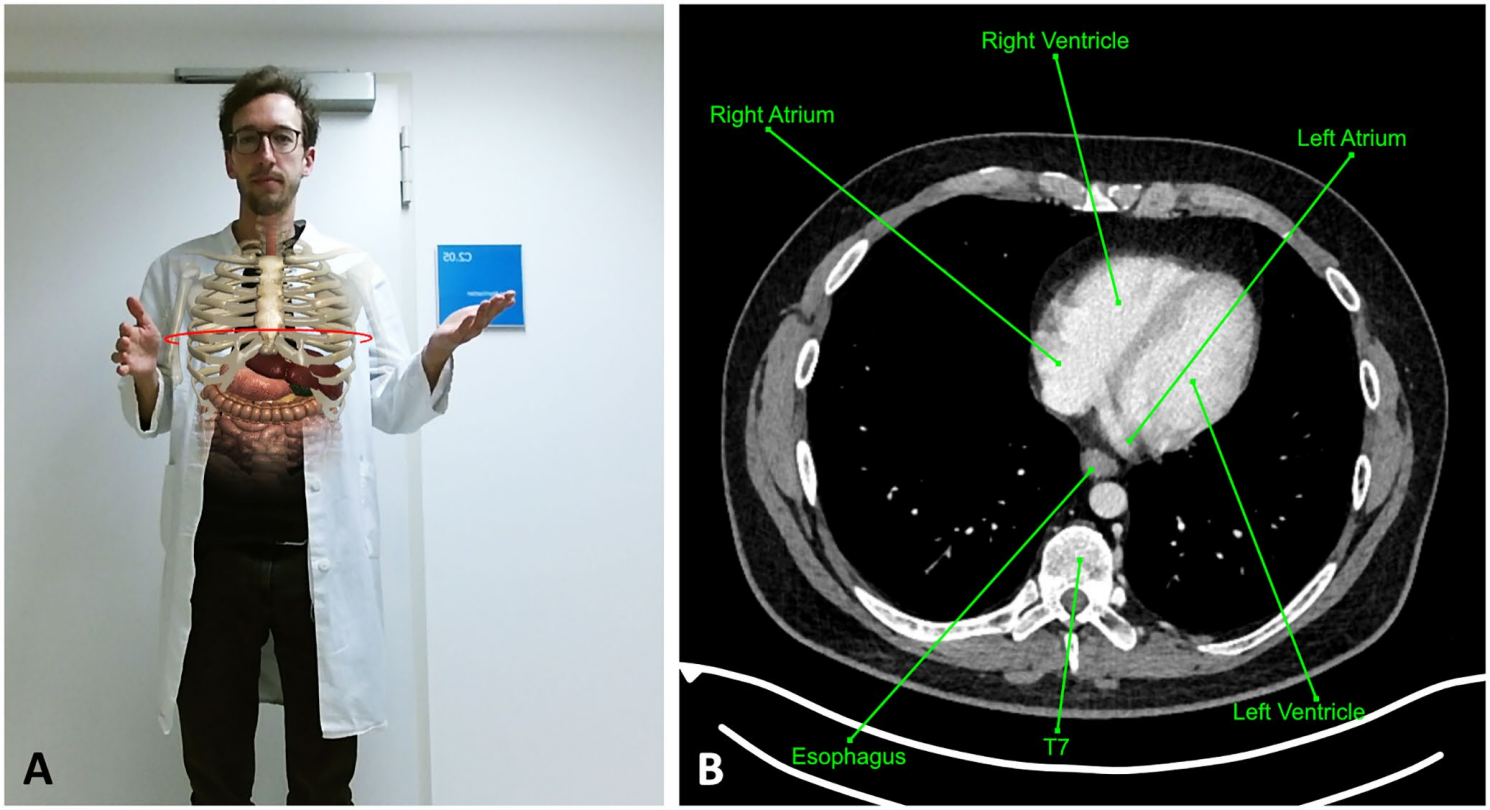
Screenshot of the magic mirror system. A, Augmented Reality (AR) view with virtual anatomy models superimposed on top of the digital mirror image of the user. B, annotated CT section image corresponding to the slice at the height of the virtual red circle in the AR view, controlled via intuitive hand gestures.

The previously proposed AR Magic Mirrors are screen‐based systems that enable users to explore anatomical structures in conjunction with medical images in relation to their own body (Blum et al., 2012; Ma et al., 2013; Stefan et al., 2014; Ma et al., 2016a, b; Bork et al., 2017a, b). A similar system has been proposed by Giraud et al. (2014) for artistically animating medical volume datasets. A Magic Mirror refers to an AR system employing the mirror metaphor, where users see a reflection of themselves with virtual information superimposed on a large display which acts as a digital representation of a mirror. For this purpose, a Microsoft Kinect (Kinect One, Microsoft, Redmond, WA) is mounted on top of the monitor and oriented toward the user. Superimposing virtual information requires accurate tracking of the users’ pose. This is achieved by the built‐in skeleton tracking algorithms offered by the Kinect platform (Kinect One, Microsoft, Redmond, WA), which provide the 3D position of a total of 25 joints in real time (Shotton et al., 2011). For the purpose of anatomy learning, the Magic Mirror provides a split screen view visualization to the students. On the right side of the screen, two‐dimensional cross‐section images of different modalities are displayed (see Figure 1). These include both CT and MRI volumes, or high‐resolution photographic images. Intuitive gestures can be used to change the image modality, switching between different section planes (axial, frontal, and sagittal), and modify the windowing in case of CT images (e.g., abdominal, lung, or bone window). Most importantly, the system allows users to explore an entire medical image volume within seconds by simply moving up and down their right hand. Medical datasets are scaled based on the height of the current user and (in case of axial images) display the one‐section image that corresponds to the current height of the users’ hand. On the left side of the screen, the users’ virtual mirror image from the Kinect color camera is displayed along with a virtual red circle indicating the height of the currently displayed section image. Furthermore, a high‐resolution 3D model is superimposed on top of the user, creating the illusion of looking inside the body and seeing the internal anatomy. The AR Magic Mirror does not require any user calibration and is ready to use as soon as a user steps in front of the system. The Magic Mirror is not commercially available and developed as a research project at the Technical University of Munich. The hardware components include a display device, a computer, and the tracking camera, which total the costs of approximately €1000 (or USD $1138). In contrast to traditional radiology atlases and all previously mentioned integration approaches, the AR Magic Mirror system facilitates the mental mapping process by providing an in situ virtual mirror visualization of medical images. In a recent study, the feasibility of the system for radiology education was demonstrated during a gross anatomy course (Kugelmann et al., 2018). To the author’s knowledge, the Magic Mirror is the only AR system to date that to be successfully integrated into such a large scale, educational setting for anatomy learning.

### Anatomage Table

**Figure 2:**
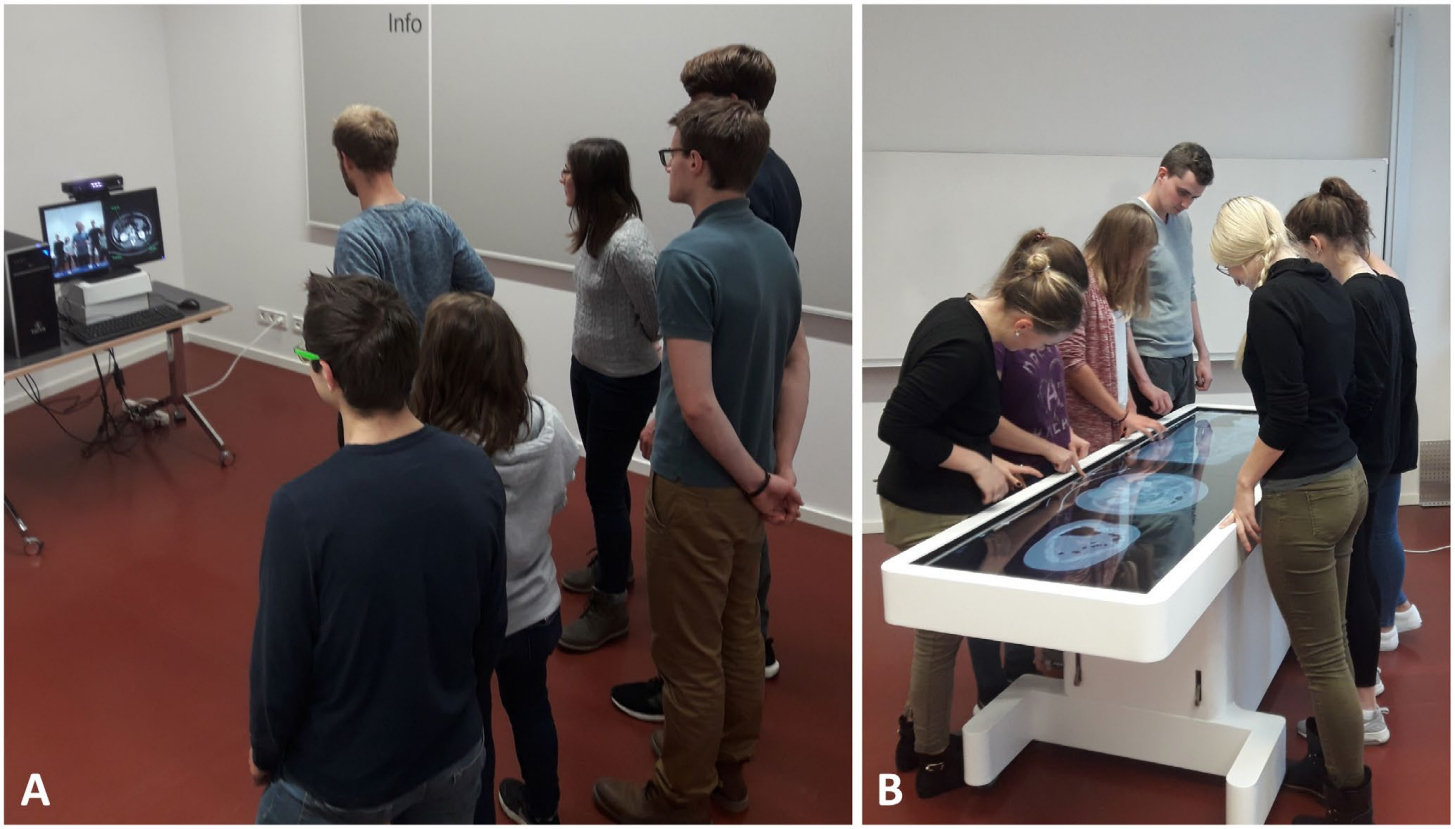
Two groups of medical students at the Ludwig‐Maximilians University in Munich interacting with A, the Magic Mirror and B, Anatomage table in a laboratory environment.

Novel radiology teaching systems such as the AR Magic Mirror have to prove their additional value in comparison to existing technologies present in radiology teaching environments. The Anatomage table (Anatomage Inc., San Jose, CA) is one specific virtual dissection table that was used as a benchmark for comparison with the AR Magic Mirror. Anatomage tables have been integrated into gross anatomy courses and their impact on students’ learning and perception has been demonstrated (Dahl and Simonsen, 2013; Eickmeyer et al., 2013; Lewandowski et al., 2013; Brown et al., 2015; Chung et al., 2015; Hutchins, 2017), even suggested as a replacement for cadavers in dissection courses (Fyfe et al., 2013; Anand and Singel, 2014; Fyfe et al., 2018), and for radiology education (Custer and Michael, 2015; Paech et al., 2017, 2018). Recently, the Anatomage was used in a clinical setting for planning of maxillofacial surgery (Brucoli et al., 2018). Anatomage is operated using touch input and allows users to interactively control a life‐sized, realistic visualization of the 3D human anatomy. Similar to the Magic Mirror, different cross‐sectional images can be displayed and investigated quickly by scrolling through the slices using the Anatomage touch table interface. All three section planes can be visualized and annotations for some anatomical structures are available. The system provides preinstalled medical image volumes including CT, MRI, as well as photographic images of cryosections. Furthermore, it is possible to upload image volumes of real patients and display them on the large LCD screen. Both Anatomage and Magic Mirror can use exactly the same cross‐section images and annotations to have comparable data. Preparation of Anatomage involves starting the application on the device and selecting the desired medical image dataset. Compared to the Magic Mirror, the costs of the Anatomage table are high (€80,000 approximately USD $91,000), as it comprises two merged high‐resolution, life‐sized touch screen displays and a computer in one housing. Figure 2 shows a group of students interacting with both the Magic Mirror and the Anatomage in a laboratory environment.

This article aims to quantitatively compare the Magic Mirror and the Anatomage Table with respect to their potential use as an additional teaching tool for radiology during a human gross anatomy course. Objective student learning outcomes were measured in both technologies and compared to learning outcomes achieved with standard radiology atlases. The authors hypothesize that all three learning modalities offer a comparable knowledge transfer and that both Anatomage and Magic Mirror are perceived as valuable additions to the gross anatomy course. Additionally, the Magic Mirror was expected to offer unique benefits to students, in particular concerning improved three‐dimensional understanding as section images are presented in direct relation to the body of the user.

## Materials and Methods

The Magic Mirror and an Anatomage virtual dissection table were integrated into dedicated tutorial sessions of a gross anatomy course over the period of one year and into an elective course for further investigation of quantitative effects.

Student data were evaluated anonymously and with permission from the students. Institutional review board approval and written informed consent were not required because all data presented in this manuscript were acquired in the course of quality assurance/quality improvement (QA/QI) measures at the institute of anatomy and cell biology at the University of Munich. Furthermore, all anatomical and radiological slice images used during this study were taken with the permission of the persons and all body donors had given their consent to donate their bodies after death for medical education and research according to international ethical guidelines and according to German law.

### Study I – Gross Anatomy Course

At the Ludwig‐Maximilians University, the education for gross anatomy is divided into both a theoretical component, teaching students materials during traditional lectures (90 hours), and a practical laboratory component which includes a compulsory dissection course (72 hours). A total number of 749 first‐year medical students took part in the gross anatomy course in winter semester 2016/17 and summer semester 2017. The dissection component is divided into five parts with the following topics: (1) Thorax and Neck; (2) Musculoskeletal System Part I; (3) Head; (4) Musculoskeletal System Part II (topography); and (5) Abdominal and Pelvis cavity. In general, 36 students dissect one body donor over the course of the laboratory in smaller groups of 12. To integrate clinical contents, five case‐based tutorial sessions were designed and integrated into the laboratory where students were able to transfer their previously acquired theoretical and dissection‐based anatomical knowledge to clinically relevant applications. For this purpose, both the Magic Mirror system and the Anatomage were used to display annotated section images of a CT volume as well as high‐resolution photographs of cryosections to facilitate this knowledge transfer. The sessions were dedicated to various anatomical topics (pelvis, shoulder, chest, abdomen, and extremities) and were adapted to the subjects currently being taught in the gross anatomy course. Each tutorial was designed to reflect a specific clinical case. Students were asked to locate relevant structures using both Anatomage and Magic Mirror, thereby introducing students to the functionalities the two systems provide. Subsequently, there was time to freely work with the systems in order to evaluate their benefits with respect to anatomy learning.

Current research on best practices for radiology education suggests that small group learning is the preferred method for both students and residents (Phillips et al., 2018). Thus, students worked in small groups (maximum six students on one device) and had the chance to interactively explore the relevant information of the various clinical contents with each system. These small group sizes ensured that each individual student had quality interaction time with both systems during the tutorials. Each student participated in one tutorial session over the period of the entire gross anatomy course. All tutorials were held by senior medical students who already finished their anatomical education, were well versed in the usage of both systems, and who received an introductory seminar to ensure that all tutors provide the same level of guidance during the tutorials. The 2‐hour tutorial was divided into a Magic Mirror and an Anatomage part as well as a short introductory explanation of the two systems and their functionalities.

#### Participants

A total number of 749 first‐year medical students took part in the tutorial sessions during the gross anatomy course in winter semester 2016/2017 (*N* = 481, 161 males, 320 females) and summer semester 2017 (*N* = 268, 105 males, 163 females). The mean age of participants was 21.0 ± 4.0 years, ranging from 18 to 35 years. All medical students were unpaid volunteers.

#### Survey

To qualitatively compare the students’ subjective attitude concerning effectiveness of both the Anatomage and the Magic Mirror system as an additional teaching resource for anatomy learning, students were asked to fill an evaluation form with 22 explicit statements concerning the usability and benefits of both systems. The survey was designed by medical education experts and all statements were tailored to provide clear and unambiguous information about the system’s capabilities. A visual analog scale (VAS) from 0 (strongly disagree) to 20 (strongly agree) for each statement was employed and users provided their approval for each statement once for the Magic Mirror (11 statements) and once for the Anatomage (11 statements). At the end of the evaluation, the survey offered a free text comment section to outline possible advantages in detail as well as criticism and potential for improvements. Filling out all questionnaires was anonymous and it was the student’s free decision to participate.

### Study II – Elective Anatomy and Radiology Course

In addition to the previously mentioned study during the gross anatomy course, the quantitative benefits and the learning effects provided by both of the two systems were analyzed during a second study which was conducted during a follow‐up elective course. During a 3‐hour, self‐directed learning session, students worked in small groups with either the Magic Mirror, Anatomage, or radiology atlases. Two multiple choice tests, before and after the learning session, were evaluated to measure the quantitative learning effect in all three groups.

#### Participants

For the elective anatomy and radiology course, a total of 72 first‐year medical students of the regular gross anatomy course were recruited from a cohort who were aiming to supervise students of the dissection course in the upcoming semester. The average age of participants was 21.36 ± 3.40 years (23 male and 49 female students), ranging from 18 to 31 years. Equivalently to the gross anatomy course, all students participated voluntarily in the elective.

#### Pretest: Anatomy knowledge and mental rotation test

**Figure 3:**
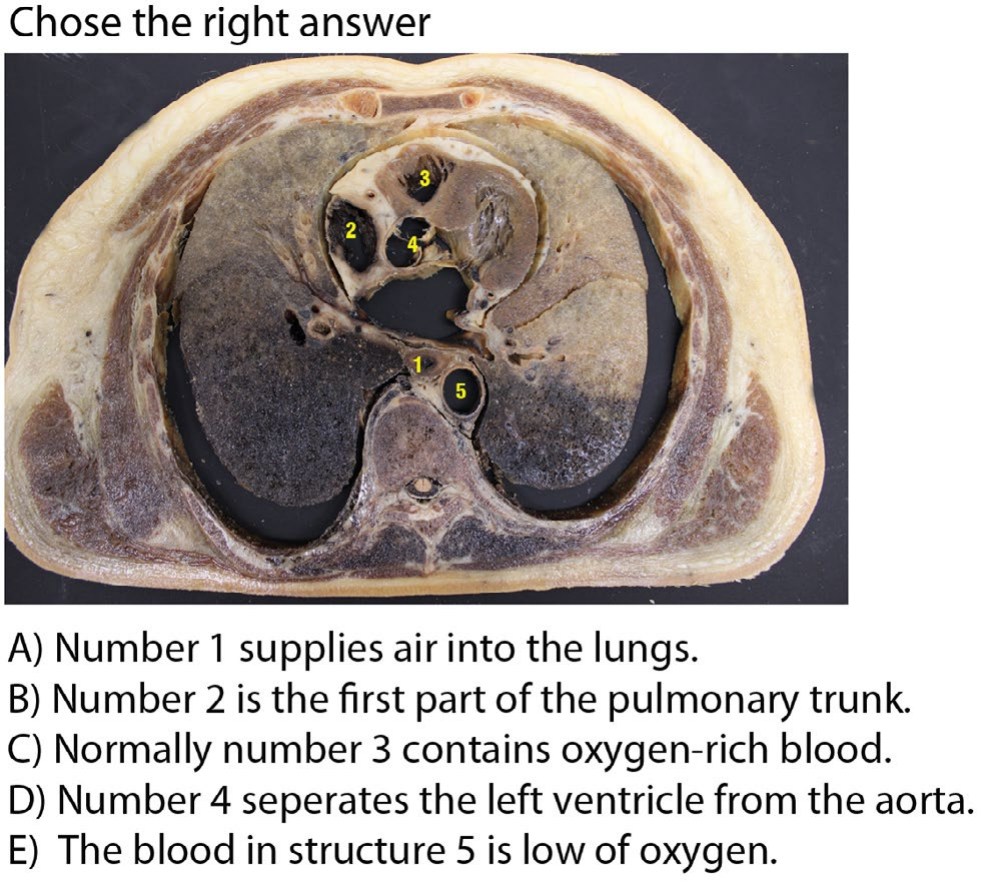
Exemplary multiple choice question from the pretest, with only one answer (D) being correct.

At the beginning of the elective course, students were asked to complete a non‐announced examination with 20 multiple choice questions similar to the anatomy part of the first main German medical examination. All questions counted equally such that the maximum number of achievable points was 20. While all questions were related to topographic anatomy, questions could either be phrased purely using text sentences (text questions) or refer to radiological or section images (image questions). All questions either consisted of statements whose correctness had to be evaluated or of positively and negatively formulated statements with only one of them being correct. Figure 3 illustrates an exemplary question aimed toward understanding of the topographic anatomy of the thorax. The questions of the pretest were categorized into the learning taxonomy of Bloom (1956). The test featured questions from two taxonomic levels of difficulty distributed equally as either “Knowledge” (10 questions) and “Comprehension” (10 questions). For the former, students should be able to retrieve, recognize, and recall relevant knowledge from memory. The latter means that students are able to construct meaning from oral, written, and graphic messages through interpreting, exemplifying, summarizing, interfering, comparing, and explaining. The reliability of the pretest was acceptable regarding the test using Cronbach’s alpha (*α* = 0.77). Students had 30 minutes to answer all questions and there were five multiple choice options to answer.

Because all participants just finished their anatomical education in gross anatomy class the questions were quite challenging to avoid a systemic bias.

In addition, a MRT was used to assess the mental rotation ability of participants. For this task, a subset of 15 pairs of 3D, freely available Shepard and Metzler‐like block stimuli images proposed by Ganis and Kievit were randomly selected from the 48 available stimuli and presented to participants (Shepard and Metzler, 1971; Ganis and Kievit, 2015). Each stimulus consisted of a combination of 7 to 11 computer‐generated cubes, composed of four arms pointing in different directions. Participants were given 1 minute to attempt to complete the task of deciding whether the 15 pairs of block stimuli were identical or mirror images of each other. In each test pair, an exemplar image is presented next to the second shape. The second shape was rotated by either 0, 50, 100, or 150 degrees with respect to the first shape. All students received a unique code after the pretest which allowed them to login to a website containing the MRT.

#### Participant sorting

Based on the results of the pretest and the MRT results, participants were sorted into three comparable groups for the subsequent, self‐directed learning session with 24 participants in each group: (1) Magic Mirror (7 males, 17 females, mean age 21.52 ± 4.38); (2) Anatomage (9 males, 15 females, mean age 21.36 ± 2.61); or (3) Theory (learning with atlases, 7 males, 17 females, mean age 21.19 ± 2.94). The participant sorting was conducted in a way, such that the average pretest and MRT results, as well as the standard deviation, were as similar as possible in all three groups (compare first columns in Tables 1 and 2). Finally, all participants received their personal pretest results and it was communicated that there will be another test after the learning session.

**Table 1:** Pre‐ and Posttest Scores of the Elective Anatomy and Radiology Course

Condition	Pretest			Posttest		
	All Questions	Image Questions	Text Questions	All Questions	Image Questions	Text Questions
	(M = 20)^a^	(M = 10)^a^	(M = 10)^a^	(M = 20)^a^	(M = 10)^a^	(M = 10)^a^
	Mean % (±SD)	Mean % (±SD)	Mean % (±SD)	Mean % (±SD)	Mean % (±SD)	Mean % (±SD)
Magic Mirror	48.00 (±13.07)	29.60 (±18.37)	54.13 (±15.43)	56.00 (±14.08)	64.89 (±19.69)	48.00 (±17.32)
(*N* = 24)						
Anatomage	48.00 (±14.22)	28.80 (±21.66)	54.40 (±17.18)	55.16 (±10.97)	59.11 (±14.60)	51.60 (±18.18)
(*N* = 24)						
Theory	50.60 (±12.53)	30.40 (±14.28)	57.33 (±16.67)	59.16 (±14.28)	59.11 (±16.89)	59.20 (±21.39)
(*N* = 24)						
All Participants	48.87 (±13.17)	29.60 (±18.12)	55.29 (±16.28)	56.77 (±13.13)	61.04 (±17.17)	52.93 (±19.37)
(*N* = 72)						

Percentages of correct answers are provided for all participants combined and for each of the three groups individually, as well as for the two types of questions (image‐based and text‐based) and all questions combined;

a Number of questions in each group.

**Table 2:** Mental Rotation Test (MRT) Scores and Improvement Percentages Between Pre‐ and Posttests for the Magic Mirror, Anatomage, and Theory Group

Condition	Entire Group (*N* = 24)		MRT – High (*N* = 12)		MRT – Low (*N* = 12)	
	MRT Score	Improvement	MRT Score	Improvement	MRT Score	Improvement
	Mean % (±SD)	Mean % (±SD)	Mean % (±SD)	Mean % (±SD)	Mean % (±SD)	Mean % (±SD)
Magic Mirror	71.80 (±22.74)	8.00 (±13.73)	91.54 (±7.38)	7.89 (±14.07)	50.42 (±10.48)	7.49 (±13.56)
Anatomage	71.88 (±20.16)	7.16 (±15.62)	87.07 (±10.28)	2.85 (±15.79)	52.55 (±10.19)	10.91 (±14.40)
Theory	71.68 (±20.71)	8.58 (±11.68)	87.21 (±9.66)	13.00 (±10.93)	51.92 (±11.84)	3.46 (±10.49)

Results are presented both for the entire group and for the two subgroups with high and low Mental Rotation Test scores individually; MRT, Mental Rotation Test.

#### Learning phase in groups

For the self‐directed learning sessions, participants gathered in spatially separated rooms where the different media were prepared: the first one with two Magic Mirror systems, the second one with two Anatomage tables, and the third room with an adequate number of anatomical and radiological atlases (Netter, 2011; Paulsen and Waschke, 2014). Prior to the learning session, it was communicated to all groups that the present tutors would solely give technical or operational support. Based on the contents queried in the pretest, a set of main topics was defined for the students to focus on during the 3 hours self‐directed learning phase, including “anatomical relations of the abdominal region,” “anatomy of the heart,” and “topography of the thorax.” These objectives concerning broader anatomical regions were chosen in favor of explicit learning statements, such as identification of certain structures, to avoid a knowledge bias in the posttest.

#### Final test

After the self‐directed learning of the declared main topics and a break of 30 minutes, participants were assessed again with a final knowledge test. This test had the same construction as the pretest but all questions were either entirely different or at least substantially modified to avoid memory bias. Similar to the pretest, questions were sampled from the same two levels of objective from Bloom’s taxonomy, with one more question (11) from the slightly more challenging “Comprehension” level and one questions less (9) from the “Knowledge” domain (Bloom, 1956). The reliability of the final test was good, tested again by Cronbach’s alpha (*α* = 0.82). All mentioned tests were not relevant for the official grading of the students and the final scores were calculated as the number of correctly answered questions in each test, respectively. For comparing the test scores of both the pre‐ and posttest examination as well as the qualitative data from both surveys, a univariate analysis of variances (ANOVA) with repeated measures in conjunction with Tukey’s post hoc tests was employed to reveal significant differences between the three different groups (Magic Mirror, Anatomage, Atlas‐based Theory). The SPSS statistical package, version 24.0 (IBM Corp., Armonk, NY), was used for the statistical analysis.

#### Extended learning session and survey

In order to compare students’ subjective attitude concerning the effectiveness of both the Anatomage and the Magic Mirror compared to traditional anatomy learning with atlases and text books after the previous more in‐depth learning sessions, students had the opportunity to work in groups with all three media in supervised learning sessions on the second day of the elective course. This way, all students were worked with all three media for at least 3 hours. At the end of day two, students were asked to fill out a final evaluation sheet to judge the quality of all three media for anatomical and medical education. The survey was exactly the same as the one executed during the gross anatomy course.

## Results

### Study I – Gross Anatomy Course

#### Survey

The results of the VAS survey data obtained during the 1‐year gross anatomy course are summarized in Table 3. Students signaled their approval to 22 explicit statements (S1 – S22) concerning the usability and additional teaching value of both Anatomage and Magic Mirror on a 20‐scale VAS. The Magic Mirror achieved comparable scores to Anatomage with slightly higher ratings for the latter for almost all statements. Both systems were found to offer comparable benefits to dissection courses (S5 – S6, *F*(1,1496) = 3.29, *P* = 0.07, ns) and greatly enhance them (S3 – S4), with significant higher scores for the Anatomage (*F*(1,1496) = 32.96, *P* < 0.001, *η* ^2^ = 0.02). However, both systems were considered not suitable for replacing dissection courses completely (S1 – S2, *F*(1,1496) = 35.31, *P* < 0.001, *η* ^2^ = 0.02). The Magic Mirror was considered significantly more intuitive to work with than Anatomage (S7 – S8, *F*(1,1496) = 26.90, *P* < 0.001, *η* ^2^ = 0.02) and both systems received good scores in terms of engineering quality (S9 – S10, *F*(1,1496) = 23.58, *P* < 0.001, *η* ^2^ = 0.02). While the Anatomage was found to be the significantly superior tool for a first contact to anatomy (S11 – S12, *F*(1,1496) = 214.86, *P* < 0.001, *η* ^2^ = 0.13), the vast majority of students could imagine working with both of the systems on their own (S13 – S14), again with significantly higher scores for Anatomage (*F*(1,1496) = 18.19, *P* < 0.001, *η* ^2^ = 0.01). Comparably, good results were obtained for the improvement of students’ subjectively assessed spatial understanding (S15 – S16, *F*(1,1496) = 3.16, *P* = 0.08, ns) as well as their anatomical knowledge (S19 – S20, *F*(1,1496) = 3.59, *P* = 0.06, ns). Significantly higher scores were obtained for the Anatomage with respect to its potential for increasing anatomical knowledge (S17 – S18, *F*(1,1496) = 21.83, *P* < 0.001, *η* ^2^ = 0.01). In terms of advantages of the two systems over traditional textbooks (S21 – S22), the Anatomage achieved significantly higher scores compared to Magic Mirror (*F*(1,1496) = 48.80, *P* < 0.001, *η* ^2^ = 0.03).

**Table 3:** Survey Results from Medical Students Comparing the Magic Mirror and Anatomage with Respect to Their Additional Value After Both the Gross Anatomy Course and the Elective Anatomy and Radiology Course

Survey Statements	Visual Analog Scale^a^			
	Gross Anatomy course		Elective Course	
	(*N* = 749)		(*N* = 72)	
	Mean % (±SD)	*P*‐value (effect size)	Mean % (±SD)	*P*‐value (effect size)
1. Magic Mirror is able to fully replace dissection courses	3.85 (±4.28)	<0.001 (S)	3.95 (±4.57)	N.S.
2. Anatomage is able to fully replace dissection courses	5.32 (±5.23)		4.86 (±4.80)	
3. Magic Mirror is a good enhancement for dissection courses	13.93 (±5.33)	<0.001 (S)	14.56 (±5.22)	N.S.
4. Anatomage is a good enhancement for dissection courses	15.46 (±4.95)		14.51 (±5.12)	
5. Magic Mirror offers no benefits to dissection courses	7.36 (±5.56)	N.S.	6.29 (±4.97)	N.S.
6. Anatomage offers no benefits to dissection courses	6.84 (±5.44)		6.74 (±5.30)	
7. Magic Mirror is intuitive to work with	14.18 (±4.71)	<0.001 (S)	16.29 (±3.82)	<0.001 (L)
8. Anatomage is intuitive to work with	12.89 (±4.90)		10.97 (±5.07)	
9. Magic Mirror seems to be well‐engineered	12.20 (±4.64)	<0.001 (S)	13.01 (±4.19)	N.S.
10. Anatomage seems to be well‐engineered	13.37 (±4.73)		11.74 (±5.31)	
11. Magic Mirror provides a good first contact to anatomy	11.52 (±3.67)	<0.001 (M)	9.68 (±6.02)	N.S.
12. Anatomage provides a good first contact to anatomy	14.84 (±5.22)		10.56 (±6.33)	
13. I can imagine working with the Magic Mirror myself	14.95 (±5.21)	<0.001 (S)	16.03 (±4.98)	<0.05 (S)
14. I can imaging working with the Anatomage myself	16.00 (±4.60)		14.32 (±5.47)	
15. Magic Mirror enhances my 3D understanding	14.36 (±4.96)	N.S.	15.32 (±3.99)	N.S.
16. Anatomage enhances my 3D understanding	14.81 (±4.80)		14.92 (±4.52)	
17. Magic Mirror can be beneficial for increasing my anatomical knowledge	13.60 (±4.84)	<0.001 (S)	15.07 (±4.44)	N.S.
18. Anatomage can be beneficial for increasing my anatomical knowledge	14.74 (±4.59)		14.72 (±4.52)	
19. Using Magic Mirror increased my personal anatomical knowledge	11.58 (±5.21)	N.S.	16.04 (±3.70)	N.S.
20. Using Anatomage increased my personal anatomical knowledge	12.09 (±5.29)		15.60 (±4.69)	
21. Magic Mirror offers advantages over traditional atlases / textbooks	11.13 (±4.91)	<0.001 (S)	12.50 (±4.93)	N.S.
22. Anatomage offers advantages over traditional atlases / textbooks	12.89 (±4.86)		12.57 (±4.93)	

a Visual Analog Scale (0 – 20), where 0 = completely disagree and 20 = completely agree; Effect sizes are indicated as (S) = small (*η* ^2^ < 0.02), (M) = medium (*η* ^2^ > 0.13), and (L) = large (*η* ^2^ < 0.26); N.S. = no statistically significant.

### Study II – Elective Anatomy and Radiology Course

#### Pretest vs. Posttest scores

In all three study groups, participants achieved higher posttest scores. For all participants combined, the scores were 48.87 ± 13.17% during the pretest, whereas during the posttest results increased to 56.77 ± 17.17%. These differences were significant at the *P < *0.001 level (*F*(1,148) = 13.56, *η* ^2^ = 0.08). For the individual three groups, participants achieved significantly higher scores during the posttest in both the Magic Mirror group (*F*(1,48) = 4.34, *P* < 0.05, *η* ^2^ = 0.08) as well as in the Theory group (*F*(1,48) = 5.08, *P* < 0.05, *η* ^2^ = 0.10). However, there were no significant differences between pre‐ and posttest scores for participants in the Anatomage group (*F*(1,48) = 3.97, *P* = 0.52, ns). The results are summarized in Table 1.

**Figure 4:**
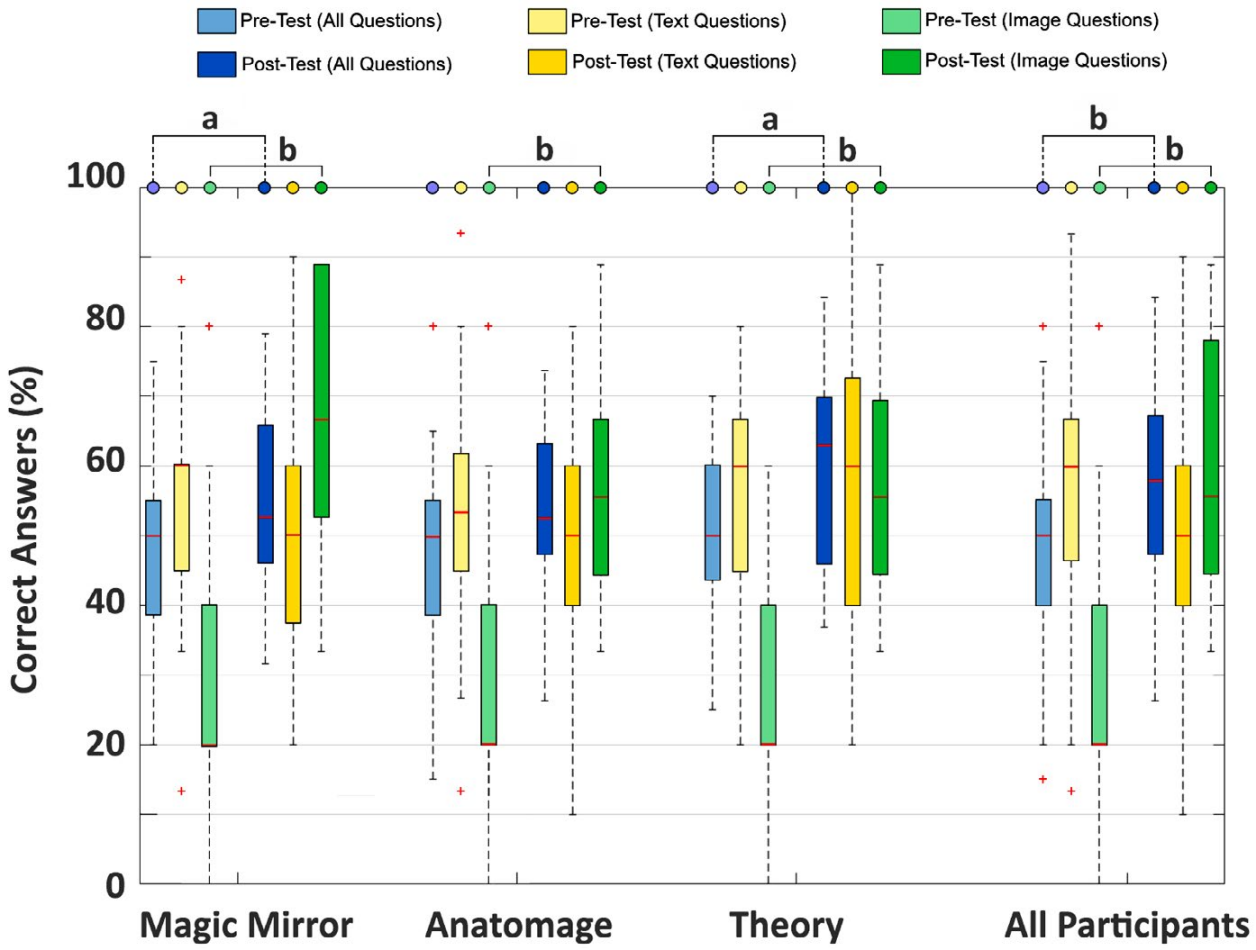
Percentages of correct answers achieved by students during both the pre and posttest. Questions could be classified either as image or text questions. Results are presented for each of the three groups (Magic Mirror, Anatomage, Atlas‐based Theory) individually as well as combined. Significant differences are indicated as ^a^ *P* < 0.05; ^b^ *P* < 0.001.

To gain more insight into the results of the individual groups, overall test scores were split into two different groups according to the two types of question that were asked. As earlier, questions in the pre‐ and posttest were taken from two types: the first type of questions (image questions) were asked in reference to given anatomical slice questions, while the second type of questions (text questions) were words targeted a general understanding of the anatomy. The results demonstrate, that the overall increase in posttest scores were resulting from a better performance for the image questions in all three groups. For the Magic Mirror, test scores increased from 29.60 ± 18.37% to 64.89 ± 19.69% (*F*(1,48) = 42.94, *P* < 0.001, *η* ^2^ = 0.47). Similarly, participants in the Anatomage group improved from 28.80 ± 21.66% to 59.11 ± 14.60% (*F*(1,48) = 33.65, *P* < 0.001, *η* ^2^ = 0.41), while for the Theory group, test scores increased from 30.40 ± 14.28% to 59.11 ± 16.89% (*F*(1,48) = 42.13, *P < *0.001, *η* ^2^ = 0.47). For the text questions, participants achieved slightly lower score changes in the posttest in both the Magic Mirror (54.13 ± 15.43% compared to 48.00 ± 17.32%) and Anatomage group (54.40 ± 17.18% compared to 51.60 ± 18.18%), while a slight decrease could be observed for the Theory group (57.33 ± 16.67% compared to 59.20 ± 21.39%). However, the differences were not significant. Figure 4 illustrates the combined results for both pretest and posttest scores as well as for the two classes of questions individually.

#### Mental rotation test analysis

According to the participant sorting, the 24 students in each of the three groups not only had similar test score results in the pretest, but also comparable mental rotation skills (Magic Mirror: 71.80 ± 22.74%, Anatomage: 71.88 ± 20.16%, Theory: 71.68 ± 20.71%). In order to analyze the influence of participants’ mental rotation ability on improvement percentages between pre‐ and posttests, a median split separating students in subgroups with high and low MRT scores (MRT scores) was performed at 70%, cf. Table 2.

For the MRT – High subgroup the following average MRT scores resulted: Magic Mirror (91.54 ± 7.38%), Anatomage (87.07 ± 10.28%), and Theory (87.21 ± 9.66%). In the MRT – Low subgroup, the average MRT scores were 50.42 ± 10.48% for Magic Mirror, 52.55 ± 10.19% for Anatomage, and 51.92 ± 11.84% for the Atlas‐based Theory group. The two subgroups (MRT – High and MRT – Low) were balanced for all three learning modalities and both contained 12 participants.

For the improvement percentage between pre‐ and posttest scores, an interesting difference between these two subgroups could be observed. In the Theory group, students with a high MRT score improved significantly more in the posttest than students with a low MRT score (13.00 ± 10.93% vs. 3.46 ± 10.49%, *F*(1,23) = 6.29, *P* < 0.05, *η* ^2^ = 0.21). The opposite effect was observed for the Anatomage, such that students with a low MRT score achieved higher improvement percentages compared to students with a high MRT score (2.85 ± 15.79% vs. 10.91 ± 14.40%). However, results were not significant in this case (*F*(1,23 = 1.60), *P* = 0.22, ns). For the Magic Mirror, all students achieved approximately the same improvement scores independent of their MRT scores (7.89 ± 14.07% vs. 7.49 ± 13.56%, *F*(1,23) = 0.001, *P* = 0.97, ns).

#### Survey

At the end of the elective course, all students were asked to fill out the same survey previously conducted during the 1‐year gross anatomy course. Table 3 depicts the results from all 72 students comparing the Magic Mirror and Anatomage. While the Anatomage seemed to be the students’ preferred choice for self‐directed anatomy and radiology learning during the first survey, the Magic Mirror outperformed the Anatomage in terms of approval rates in almost all statements during the second survey.

Similar to the previous survey, both systems were considered a valuable addition for enhancing dissection courses (S3 – S4, *F*(1,142) = 0, *P* = 0.96, ns and S5 – S6, *F*(1,142) = 0.27, *P* = 0.6, ns), albeit not as a full replacement (S1 – S2, *F*(1,142) = 1.3, *P* = 0.25, ns). The results for statements S7 – S8 demonstrate that the Magic Mirror has clear advantages over Anatomage in terms of intuitiveness. The VAS scores were significantly higher for the Magic Mirror at the *P* = 0.001 level (*F*(1,142) = 50.53, *P* = 0.25, *η* ^2^ = 0.26). Compared to the survey data from the gross anatomy course (Study I) to the elective course (Study II), approval levels increased for the Magic Mirror from 14.18 ± 4.71 to 16.29 ± 3.82, while they dropped for Anatomage from 12.89 ± 4.90 to 10.97 ± 5.07. In contrast to the survey from Study I, the Magic Mirror was considered to be the better‐engineered tool (S9 – S10). However, the differences were not statistically significant (*F*(1,142) = 2.57, *P* = 0.11, ns). While Anatomage was considered a great tool for the first contact to anatomy in the first survey (S11, 14.84 ± 5.22), these results could not be confirmed during Study II and a slight drop to 10.56 ± 6.33 was observed. The VAS scores also decreased for the Magic Mirror from 11.52 ± 3.67 to 9.68 ± 6.02 (S12). No significant differences were recorded between the two (*F*(1,142) = 0.72, *P* = 0.4, ns). Compared to the Study I survey, even more students could imagine working with the Magic Mirror during self‐directed learning sessions after finishing the elective course (S13, 14.95 ± 5.21 vs. 16.03 ± 4.98) while scores decreased for Anatomage (S14, 16.00 ± 4.60 vs. 14.32 ± 5.47). The VAS scores for self‐directed learning (S13 – S14) were significantly higher than those of Anatomage (*F*(1,142) = 5.06, *P* = 0.03, *η* ^2^ = 0.03). Furthermore, the Magic Mirror was found to increase 3D understanding (S15 – S16) and the personal knowledge about the anatomy slightly more than Anatomage (S15 – S20). However, none of these differences were statistically significant (S15 – S16: *F*(1,142) = 0.71, *P* = 0.4, ns; S17 – S18: *F*(1,142) = 0.04, *P* = 0.84, ns; S19 – S20: *F*(1,142) = 0.4, *P* = 0.51, ns). Lastly, students found both systems to offer almost the same benefits over traditional atlases and textbooks (S21 – S22, *F*(1,142) = 0.1, *P* = 0.93, ns).

#### Student perceptions

As part of the second survey, students could provide written feedback in free text fields about their subjective perceptions on the usage of both systems during the elective course. A total of 57 students (79.17%) took this opportunity in addition to answering the 22 survey statements. Overall, the written feedback was consistent with the quantitative statement data results. Both the Magic Mirror (*n* = 13) and Anatomage (*n* = 11) were considered great tools for increasing the 3D understanding of topographic anatomy. Both systems were found to “offer a better way of learning section images than text books,” “improve the understanding of the relative position of organs in the body,” and “increase my spatial understanding.” Furthermore, the two systems allowed to “quickly explore an entire 3D volume,” “jump to certain structures much faster [than radiology atlases],” and “easily trace the course of vessels.”

Five students explicitly appreciated the possibility of the Magic Mirror to “show anatomy on my own body,” which was found to “improve my three‐dimensional understanding” and to “help me understand at what height certain anatomical structures are located.” Two other common themes as to why some students appreciated working with the Magic Mirror were the interactive user interface (*n* = 9) and the possibility for self‐directed learning (*n* = 7). Regarding the first point, students appreciated “the very intuitive user interface and user interaction,” “the concise and accurate gesture control,” as well as the “[the system’s] simplicity of user interaction.” In terms of self‐directed learning with the Magic Mirror, students could imagine “working with the system at home using my own TV or laptop” and considered it “great for learning [certain anatomical concepts] on my own.” The Anatomage received positive feedback from students for its large display (*n* = 6), which was considered “great for providing a good overview of many different section images at the same time,” and for collaborative learning in small groups (*n* = 6), which “fueled discussions on topographic anatomy [between students].”

In terms of limitations, some students (*n* = 3) found the Magic Mirror “tiring to use for long learning sessions” and lacking a “multi‐user mode” (*n* = 2). For the Anatomage, negative feedback was mainly due to technical difficulties of the system, especially the “unresponsive touch display” (*n* = 8) and the “missing multi‐touch capabilities” (*n* = 3). Despite the positive feedback of both systems regarding improved 3D understanding, a large number of students stressed in their comments that none of the two systems is able to replace a dissection course (*n* = 18), especially due to “missing haptics” (*n* = 10). Two students considered the two systems “interesting toys” and “fun‐to‐play‐with systems, which cannot replace learning using text books.” Other general comments were concerned with the overall feedback of the course (*n* = 8), which was found to “increase my personal anatomy knowledge” and “offer a good repetition of topographic anatomy,” as well as potential improvement suggestions for the two systems to “include pathologies” (*n* = 4), “display more annotations” (*n* = 4), and “include quiz‐based learning” (*n* = 2).

## Discussion

The goal of the present study was to compare the performance of Anatomage and Magic Mirror for integrated radiology teaching in gross anatomy and to measure whether the two systems provide advantages over learning with radiology atlases. Three main observations may be inferred from the results of the user study.

Firstly, a positive, overall learning effect was measured in all three groups (Magic Mirror, Anatomage, Theory) during the elective anatomy and radiology course. While this outcome was certainly expected for the theory group, it confirmed previous studies that demonstrated an increased learning performance for the Anatomage (Anand and Singel, 2014; Fyfe et al., 2018). Additionally, it confirmed the initial hypothesis that the Magic Mirror also provides similar learning effects (Ma et al., 2016b; Kugelmann et al., 2018). For the Magic Mirror, these results are particularly promising as both Anatomage and traditional radiology atlases are well‐established learning modalities and novel technologies such as the Magic Mirror have to prove their additional pedagogic value in comparison to existing ones. On the other hand, the positive results verify recent studies describing the potential for interactive learning experiences, especially by means of AR and VR technology (Cheng and Tsai, 2013; Diegmann et al., 2015; Akçayır and Akçayır, 2017). The present results from the elective anatomy and radiology course indicate that interactive AR systems in fact can be incorporated successfully into medical curricula and provide an effective additional teaching device for radiology learning during a human gross anatomy course. While the positive, overall learning effect presents an interesting finding, a more detailed analysis of the improvement between pre‐ and posttest scores offers further insights into the specific benefits of all three learning modalities. Interestingly, no statistically different changes in scores were recorded for text questions regarding the topographical anatomy. However, test scores improved significantly when images were present to substantiate the topographical relations between structures subject to the test questions. The slight decline in correctly answered text questions for the Anatomage and Magic Mirror groups, in conjunction with a slight increase in those questions for the Theory group, could be explained by the additional textual information traditional radiology atlases provided with respect to topographic anatomy. While the former two systems were limited to displaying annotated section images, students were able to read textual information inside the radiology atlases which accompanied the section images and thus potentially lead to an increased knowledge. Another hypothesis that could explain the statistically nonsignificant differences in text questions could be that knowledge concerning the topographic characteristics is acquired more effectively during the dissection course, which offers unique advantages neither of the three learning modalities can provide. This would be consistent with both the results from the survey data and participants’ subjective perceptions, confirming that the Anatomage and Magic Mirror are valuable additions to a dissection course and increase the anatomical knowledge, but are not able to fully replace a dissection course. While not explicitly analyzed, the same holds true for traditional radiology atlases. Therefore, the better overall performance in the posttest was mainly due to a significant improvement in image questions for all three groups. Although all students participated in the gross anatomy course and learned about the basics of topographic anatomy during both lectures and a dissection course, pretest results showed a deficiency in questions concerned with radiological section images, indicating that creating a link between the topographic anatomy and radiological slices is difficult to achieve and requires additional teaching modalities. This is consistent with recent studies calling for more integrated radiology education in gross anatomy (Dmytriw et al., 2015; Heptonstall et al., 2016). Among the three learning modalities, students in the Magic Mirror group achieved the highest improvements for correctly answered image questions, closely followed by the Theory and Anatomage group. A potential explanation for this slight edge could be that the Magic Mirror depicts radiological slices in relation to the body of its user offering an egocentric versus an object‐centered spatial relationship advantage. This was mentioned by several participants and outlined as one of the key benefits of the Magic Mirror over the other two modalities. However, further studies are necessary to investigate whether such an in situ visualization improves the mental mapping capabilities of students.

The second observation concerns the results of the two surveys comparing Anatomage and Magic Mirror which revealed slightly inconsistent perceptions of the two systems. During the 1‐year gross anatomy course, Anatomage was considered superior to the Magic Mirror and achieved higher scores in almost all the statement categories. In contrast, the opposite was true when surveying students during the elective anatomy and radiology course. During the tutorial sessions of the former, interaction time with the two systems was limited, although students were distributed into small groups and tutors assured that every student worked with both systems. Compared to the Anatomage as an already well‐established anatomy teaching modality, the Magic Mirror was considered an interesting and fun‐to‐play‐with tool, but the immediate benefits and real use cases for enhancing students’ anatomy knowledge were less obvious. During the more intense elective course, however, acceptance of the AR Magic Mirror strongly increased and exceeded that of Anatomage in almost all parts of the survey. Many students stated that they could imagine working with the AR Magic Mirror themselves and appreciated the intuitiveness of the system, indicating that it is not only useful during dedicated learning sessions as part of the medical curriculum, but also as an additional teaching device for self‐directed learning. The differences in interaction time during the 1‐year gross anatomy course and the elective course are also likely the reason for the large variation of mean scores for survey statements S19 and S20 (Using Magic Mirror / Anatomage increased my personal anatomical knowledge) as well as the large standard deviation. Due to the time restrictions, the purpose of the tutorial sessions was primarily a direct transfer of knowledge. On the other hand, students were able to explore more freely the possibilities of the two systems during the elective course, facilitating not only the transfer but also the generation of knowledge. Overall, the survey results, in combination with students’ subjective feedback, demonstrate that both Anatomage and Magic Mirror can be valuable additions during integrated radiology teaching in gross anatomy courses. These findings are aligned with current research papers calling for supplementary teaching modalities, that are not aimed at replacing existing ones, but rather enable multimodal, self‐directed learning (Sugand et al., 2010; Singh and Kharb, 2013; Estai and Bunt, 2016; Phillips et al., 2018). Especially, interactive 3D techniques have the potential to improve the knowledge of anatomy and are increasingly demanded by medical students (Moro et al., 2017; Triepels et al., 2018). However, most modern‐day medical curricula do not incorporate these novel learning tools yet. As such, the present study is a first step into this direction by providing a quantitative evaluation of the Magic Mirror as one specific 3D learning tool and comparing its effectiveness and benefits to established anatomy learning modalities.

The third important observation is the correspondence between students’ MRT score and their improvement percentage between pre‐ and posttest. Students with a low MRT score and therefore with a poor spatial ability and three‐dimensional imagination achieved higher posttest scores in the Anatomage and Magic Mirror group than students in the Theory group. These results indicate that both systems improve the understanding of spatial relationships inside the human body, which is difficult to obtain from plain 2D projections in regular atlases and textbooks, especially for students with a low mental rotation ability. These results not only confirm the findings from both the survey analysis and students’ qualitative feedback, but also indicate that both Anatomage and Magic Mirror could be associated with facilitating the development of spatial reasoning skills in low MRT students, which will be an interesting direction for future research. Spatial ability has previously been reported to influence anatomy learning (Garg et al., 2001; Vorstenbosch et al., 2013). In a recent study, Sweeney et al. (2014) reported a weak association between anatomy examination scores and spatial ability. Rizzolo and Stewart (2006) argue that, especially the connection between dissection course and imaging modalities is responsible for developing spatial reasoning skills. Further studies are required to evaluate the effects of both Anatomage and Magic Mirror on students’ spatial ability acquisition, for example, by introducing both systems into the dissection theater and displaying radiological section images corresponding to the body directly on site, similar to recent studies by Paech et al. (2017, 2018).

Overall, both Anatomage and Magic Mirror have proven their benefits as additional teaching modalities during integrated radiology education in gross anatomy. The two systems increased student anatomical knowledge, improved 3D understanding of anatomical structures, and provided a good supplement to traditional text book learning. While both can be used effectively for small‐group learning, they only support single‐user interaction and can thus only be operated by one student at a time. One specific advantage of the Magic Mirror is concerned with its significantly lower hardware requirements, which would in principle allow the software to run at home on any consumer laptop with an integrated webcam. On the other hand, the high costs and large form factor of Anatomage only permit a usage in specialized university environments. Furthermore, as students’ preferences toward different learning modalities are very subjective, neither of the two systems is expected to perfectly fit the needs of every medical student. Results and subjective feedback from students during the studies indicate that especially those students with lower spatial reasoning skills can gain from learning with 3D technologies such as Anatomage and Magic Mirror. As AR and VR are becoming increasingly popular in education (Bacca et al., 2014; Billinghurst et al., 2014; Akçayır and Akçayır, 2017), it will be interesting to see whether 3D tools such as the Magic Mirror can make the transition from research projects to frequently used supplementary learning tools for medical students around the world.

### Limitation of the Study

There are some limitations to the studies presented in this study. First, due to time limitations and because tutorial sessions always corresponded to the topic currently being taught during the accompanying lecture, it was not possible for all medical students to attend all tutorials. Instead, each group only attended a subset of tutorial sessions (e.g. Group A: pelvis and thorax; Group B: head and neck; etc.) However, interaction with both Anatomage and AR Magic Mirror was comparable in all sessions, such that only the anatomy of interest, but not the type of interaction varied between the groups. Secondly, while identical section images were available on both the AR Magic Mirror and Anatomage, this was not possible for the Theory group. Students in the latter group used a radiology atlas during the self‐directed learning session. However, all slices of the volumetric dataset were manually labeled by experts according to the terminology in the radiology atlas, such that all relevant information to answer the questions in both pre‐ and posttest was available for all three groups. Thirdly, related to the second limitation is the fact that students had only a limited amount of section images available during the two studies. Pathologies were not part of the investigations, but could be a topic of interest for future research. Fourthly, the cognitive load associated with using Anatomage or Magic Mirror was not specifically studied. Especially in AR‐based education, novel systems should not overload the user with virtual information (Wu et al., 2013). However, the AR overlay of Magic Mirror is limited to displaying section images and a line indicating the current height of a slices and none of the participants mentioned experiencing cognitive difficulties while working with the system. Finally, the current results arise from a single‐center study and the number of participants in the elective radiology and anatomy course was not specifically large. Future studies performed in multiple centers are required to validate the results and discover other potential application scenarios for 3D tools such as Anatomage and Magic Mirror in the medical curriculum.

## Conclusion

This article presented supporting evidence that the AR Magic Mirror system provides great potential as an additional teaching device for integrated anatomy courses. The benefits were evaluated during a 1‐year gross anatomy course as well as an intense, follow‐up elective course which compared the system to the Anatomage as a state‐of‐the‐art system for combined anatomy and radiology education. A quantitative learning effect could be observed during the studies and demonstrated the systems’ benefits for engaging, interactive, and self‐directed learning. Furthermore, the system proved to be particularly powerful for increasing the spatial understanding particularly in low spatial ability students. The findings suggest that AR systems for integrated radiology teaching in gross anatomy such as the Magic Mirror offer the potential to become a unique and powerful learning tool as well as an integral part of both modern medical curricula and students’ educational toolsets.

## Notes on Contributors

FELIX BORK, M.Sc., is a graduate (Ph.D.) student in the Chair for Computer‐Aided Medical Procedures and Augmented Reality at the Technical University of Munich, in Munich, Germany. His research interest is in mixed reality for medical education and perceptual visualization.

LEONARD STRATMANN, is an undergraduate medical student in the Chair for Vegetative Anatomy, at the Faculty of Medicine, Ludwig‐Maximilians University,

Munich, Germany. His research interest is in novel teaching devices for interactive anatomy and radiology education.

STEFAN ENSSLE, is an undergraduate medical student in the Chair for Vegetative Anatomy at the Faculty of Medicine, Ludwig‐Maximilians University, Munich, Germany. His research interest is in novel didactic methods for student‐centered anatomy and radiology education.

ULRICH ECK, Ph.D., is a senior research scientist in the Chair for Computer‐Aided Medical Procedures at the Technical University of Munich in Munich, Germany, where he is managing the research activities on medical‐augmented reality. His research interests include: medical applications, real‐time systems, human‐computer interaction, and software engineering.

NASSIR NAVAB, Ph.D., is a professor and head of the Chair for Computer‐Aided Medical Procedures and Augmented Reality at the Technical University of Munich, in Munich, Germany. He is also a professor in the Laboratory for Computational Sensing and Robotics, at Johns Hopkins University in Baltimore, MD. His research interests include computer‐aided medical procedures and augmented reality.

JENS WASCHKE, Ph.D., is a professor and head of the Chair for Vegetative Anatomy at the Ludwig‐Maximilians University of Munich, in Munich Germany. He conducts research in the field of cell biology on the regulation of the endothelial barrier and cell cohesion and is one of the two editors of the Sobotta Anatomy Atlas and other textbooks on anatomy.

DANIELA KUGELMANN, Ph.D., is a postdoctoral fellow in the Chair for Vegetative Anatomy at the Ludwig‐Maximilians University of Munich, Munich, Germany. Her research is in the field of cell biology and technology‐enhanced medical education. She is managing the teaching activities at the Chair for Vegetative Anatomy and is responsible for conducting projects for improving the curricular education.
